# Bis(2,2′-bipyridyl dioxide-κ^2^ *N*,*N*′)bis­(tricyano­methanido)cobalt(II) dihydrate

**DOI:** 10.1107/S1600536810017435

**Published:** 2010-05-19

**Authors:** Li-Juan Qiu, Jun Luo, Xin-Rong Zhang, Bao-Shu Liu

**Affiliations:** a School of Pharmacy, Second Military Medical University, Shanghai 200433, People’s Republic of China

## Abstract

In the title compound, [Co(C_4_N_3_)_2_(C_10_H_8_N_2_O_2_)]·2H_2_O, a novel tricyano­methanide complex, the Co^II^ atom is located on an inversion center and has a distorted octa­hedral coordination with two 2,2′-bipyridyl dioxide (dpdo) mol­ecules and two *trans* tricyano­methanide (tcm) anions. The equatorial plane is formed by the four O atoms of the two chelating dpdo ligands, with one N atom of each of the two tcm ligands occupying an apical position. There is a disordered solvent water mol­ecule in the asymmetric unit (occupancy ratio 0.63:0.37). These water mol­ecules result in the formation of O—H⋯O and O—H⋯N hydrogen bonds, building a layer parallel to (100). The layers are linked by C—H⋯N hydrogen-bonding inter­actions, leading to a three-dimensional network.

## Related literature

For coordination polymers constructed with tricyano­methanide, see: Abrahams *et al.* (2003 ▶); Batten & Murray (2003 ▶); Batten *et al.* (1998 ▶, 1999 ▶, 2000 ▶); Feyerherm *et al.* (2003 ▶, 2004 ▶); Hoshino *et al.* (1999 ▶); Manson & Schlueter (2004 ▶); Manson *et al.* (1998 ▶, 2000 ▶); Miller & Manson (2001 ▶); Yuste *et al.* (2007 ▶, 2008 ▶). For complexes containing dpdo, see: Luo *et al.* (2009 ▶); Zhang *et al.* (2010 ▶); Su & Lan (2007 ▶).
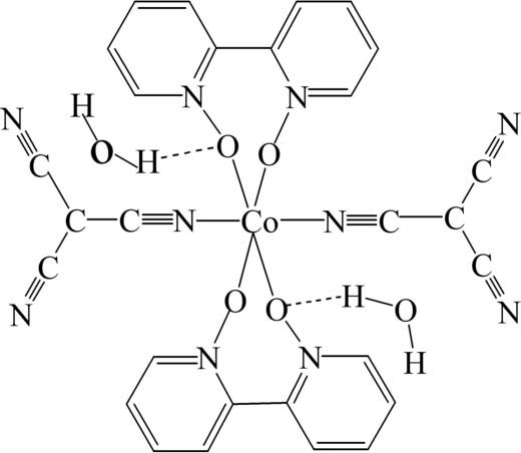


## Experimental

### 

#### Crystal data

[Co(C_4_N_3_)_2_(C_10_H_8_N_2_O_2_)]·2H_2_O
*M* *_r_* = 651.47
Monoclinic, 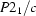

*a* = 9.575 (3) Å
*b* = 16.699 (6) Å
*c* = 9.442 (3) Å
β = 95.307 (4)°
*V* = 1503.3 (9) Å^3^
*Z* = 2
Mo *K*α radiation
μ = 0.63 mm^−1^
*T* = 293 K
0.15 × 0.12 × 0.10 mm

#### Data collection

Bruker SMART APEX CCD area-detector diffractometer
Absorption correction: multi-scan (*SADABS*; Bruker, 2000 ▶) *T*_min_ = 0.911, *T*_max_ = 0.940
7102 measured reflections
3193 independent reflections
2511 reflections with *I* > 2σ(*I*)
*R*_int_ = 0.026

#### Refinement

*R*[*F* ^2^ > 2σ(*F* ^2^)] = 0.032
*wR*(*F* ^2^) = 0.084
*S* = 0.99
3193 reflections
214 parameters
H-atom parameters constrained
Δρ_max_ = 0.27 e Å^−3^
Δρ_min_ = −0.21 e Å^−3^

### 

Data collection: *SMART* (Bruker, 2000 ▶); cell refinement: *SAINT* (Bruker, 2000 ▶); data reduction: *SAINT*; program(s) used to solve structure: *SHELXS97* (Sheldrick, 2008 ▶); program(s) used to refine structure: *SHELXL97* (Sheldrick, 2008 ▶); molecular graphics: *ORTEPIII* (Burnett & Johnson, 1996 ▶), *ORTEP-3 for Windows* (Farrugia, 1997 ▶) and *PLATON* (Spek, 2009 ▶); software used to prepare material for publication: *SHELXL97*.

## Supplementary Material

Crystal structure: contains datablocks global, I. DOI: 10.1107/S1600536810017435/dn2560sup1.cif

Structure factors: contains datablocks I. DOI: 10.1107/S1600536810017435/dn2560Isup2.hkl

Additional supplementary materials:  crystallographic information; 3D view; checkCIF report

## Figures and Tables

**Table 1:** Hydrogen-bond geometry (Å, °)

*D*—H⋯*A*	*D*—H	H⋯*A*	*D*⋯*A*	*D*—H⋯*A*
O3—H3*B*⋯O1	0.86	2.00	2.851 (8)	173
O3*B*—H3*D*⋯O1	0.86	2.09	2.890 (15)	156
O3—H3*A*⋯N4^i^	0.86	2.24	3.028 (8)	152
O3*B*—H3*C*⋯N4^i^	0.85	2.21	3.056 (15)	174
C1—H1⋯N4^ii^	0.93	2.55	3.437 (3)	161
C4—H4⋯N5^iii^	0.93	2.38	3.287 (3)	165
C10—H10⋯N4^iv^	0.93	2.48	3.390 (3)	164

Symmetry codes: (i) 
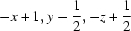
; (ii) 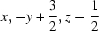; (iii) 
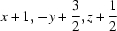
; (iv) 
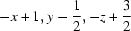
.

**Fig. 1.**
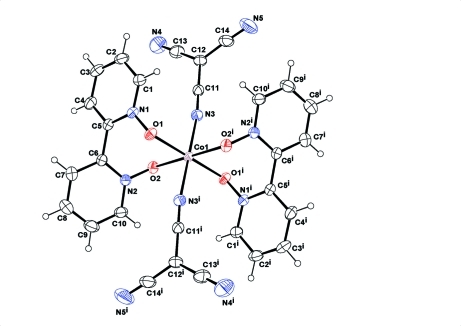
A view of the mononuclear structure in (I), showing the atom- labelling scheme. Displacement ellipsoids are drawn at the 50% probability level and H atoms have been omitted for clarity [symmetry code: (i) -x+1, -y+1,-z+1].

**Fig. 2.**
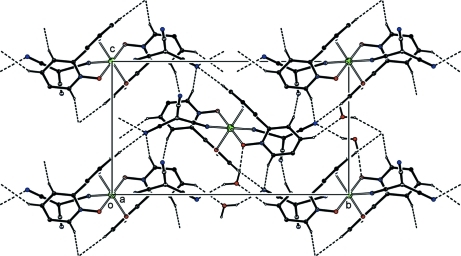
Partial packing view along the a axis showing the three dimensional structure formed through O-H···O, O-H···N and C-H···N hydrogen bondings. H atoms not involved in hydrogen bondings have been omitted for clarity.

## supplementary crystallographic information

### Comment

Coordination polymers constructed by tricyanomethanide (tcm) have attracted considerable interest due to their diverse structures and fascinating magnetic properties (Batten *et al.*, 2003; Miller *et al.*, 2001; Feyerherm *et al.*, 2003). Notably, except a doubly interpenetrated (6,3) sheet was observed in Ag(tcm)_2_ (Abrahams *et al.*, 2003), most binary tcm complexes display a rutile-like structure (Manson *et al.*, 2000, 1998; Hoshino *et al.*, 1999; Feyerherm *et al.*, 2004). To gain insight into the influence of the co-ligands on the structures and magnetic properties of tcm complexes, some co-ligands such as hexamethyl-enetetramine, 4,4-bipyridyl, 1,2-bi(4-pyridyl)ethane were introduced to the binary tcm systems. Among the Cu(I) or Cd(II) tcm complexes with these co-ligands, numerous structure types range from doubly interpenetrated (4,4) sheet to 3D rutile networks were observed (Batten *et al.*, 2000, 1998). By contrast, modification of the Mn(II)-tcm binary system with 4,4-bipyridyl as co-ligands leads to the formation of a one dimensional chain-like structure (Manson *et al.*, 2004). Recently, several copper tcm complexes with nitrogen-containing heterocyclic co-ligands has been characterized (Yuste *et al.*, 2008, 2007). On the other hand, 2,2'-dipyridyl N,*N*'-dioxide (dpdo) is a new co-ligand and has two potential oxygen donor atoms, however, few tcm complex with dpdo co-ligand has been reported (Luo *et al.*, 2009). During our systematic investigation of the nature of dpdo co-ligand on the structures and properties of tcm complexes, we obtained a new tcm complex Co(dpdo)_2_(C_4_N_3_)_2_(H_2_O)_2_ (I), we herein report the synthesis and crystal structure of the complex.

In the title compound, the cobalt atom, located on an inversion center, has a distorted octahedral geometry with two dpdo molecules and two trans tricyanomethanide. The equatorial plane being formed by the four O atoms of the two chelating dpdo ligands whereas one N atom of each tcm ligands occupying the apical positions (Fig. 1).

Interestingly, two solvate water molecules are observed and the situation is different from the similar manganese complex reported in which no water molecules were found (Luo *et al.*, 2009). In the title compound, these water molecules result in the formation of O-H···O and O-H···N hydrogen bonds building a layer parallel to the (1 0 0) plane (Table 1, Fig. 2). Furthermore, C-H···N hydrogen interactions (Table 1) link these layers forming a three dimensionnal network.

The Co—O(dpdo) distances are in the range 2.050 (1)Å - 2.070 (1) Å, these values are comparable to the corresponding distances in cobalt- nitroxide complexes (Zhang *et al.*, 2010) and in the Co(dpdo)_2_(H_2_O)_2_ (Su & Lan, 2007). The Co—N(tcm) distances are 2.110 (2) Å, and the data are similar to the corresponding distances observed in cobalt tcm complex (Batten *et al.*, 1999).

Each tricyanomethanide moiety is almost planar. Bond distances and bond angles within the anions are in good agreement with those found in other tricyanomethanide complexes (Hoshino *et al.*, 1999; Batten *et al.*, 1999).

### Experimental

A 5 ml warm acetonitrile solution of 2,2'-dipyridyl N,*N*'-dioxide (0.10 mmol, 18.82 mg) and a 2 ml aqueous red solution of cobalt nitrate (0.10 mmol, 29.10 mg) were mixed and stirred for 5 min s, the mixed solution was orange. To the mixture was added a 3 ml acetonitrile-water solution (CH_3_CN:H_2_O = 2:1, V:V) of potassium tricyanomethanide (0.20 mmol, 25.83 mg). After stirred for another 5 min s, the orange solution was filtered and the filtrate was slowly evaporated in air. After two weeks, orange block crystals of I were isolated in 34% yield.

Anal: Calculated for C_28_H_20_CoN_10_O_6_: C 51.62%, H 3.10%, N 21.50%. Found C 51.77%, H 3.19%, N 21.64%.

### Refinement

All H atoms attached to C atoms were fixed geometrically and treated as riding with C—H = 0.93 Å with U_iso_(H) = 1.2U_eq_(C). H atoms of water molecule were located in difference Fourier maps and included in the subsequent refinement using restraints (O-H= 0.85 (1)Å and H···H= 1.39 (2)Å) with U_iso_(H) = 1.5U_eq_(O). In the last stage of refinement, they were treated as riding on the O atom.

The water molecules appear to be disorered over two positions with occupancy factors roughly in the ratio 2/1. The occupancy factors were determined using a constrained refinement with the sum of the occupancy fixed to 1.

### Crystal data

**Table:**

[Co(C_4_N_3_)_2_(C_10_H_8_N_2_O_2_)]·2H_2_O	*F*(000) = 666
*M**_r_* = 651.47	*D*_x_ = 1.439 Mg m^−^^3^
Monoclinic, *P*2_1_/*c*	Mo *K*α radiation, λ = 0.71073 Å
Hall symbol: -P 2ybc	Cell parameters from 887 reflections
*a* = 9.575 (3) Å	θ = 2.5–26.6°
*b* = 16.699 (6) Å	µ = 0.63 mm^−^^1^
*c* = 9.442 (3) Å	*T* = 293 K
β = 95.307 (4)°	Block, orange
*V* = 1503.3 (9) Å^3^	0.15 × 0.12 × 0.10 mm
*Z* = 2	

### Data collection

**Table:**

Bruker SMART APEX CCD area-detector diffractometer	3193 independent reflections
Radiation source: fine-focus sealed tube	2511 reflections with *I* > 2σ(*I*)
graphite	*R*_int_ = 0.026
phi and ω scans	θ_max_ = 27.0°, θ_min_ = 2.1°
Absorption correction: multi-scan (*SADABS*; Bruker, 2000)	*h* = −12→7
*T*_min_ = 0.911, *T*_max_ = 0.940	*k* = −20→18
7102 measured reflections	*l* = −8→12

### Refinement

**Table:**

Refinement on *F*^2^	Primary atom site location: structure-invariant direct methods
Least-squares matrix: full	Secondary atom site location: difference Fourier map
*R*[*F*^2^ > 2σ(*F*^2^)] = 0.032	Hydrogen site location: inferred from neighbouring sites
*wR*(*F*^2^) = 0.084	H-atom parameters constrained
*S* = 0.99	*w* = 1/[σ^2^(*F*_o_^2^) + (0.0476*P*)^2^] where *P* = (*F*_o_^2^ + 2*F*_c_^2^)/3
3193 reflections	(Δ/σ)_max_ < 0.001
214 parameters	Δρ_max_ = 0.27 e Å^−^^3^
0 restraints	Δρ_min_ = −0.20 e Å^−^^3^

### Special details

**Table:**

Geometry. All esds (except the esd in the dihedral angle between two l.s. planes) are estimated using the full covariance matrix. The cell esds are taken into account individually in the estimation of esds in distances, angles and torsion angles; correlations between esds in cell parameters are only used when they are defined by crystal symmetry. An approximate (isotropic) treatment of cell esds is used for estimating esds involving l.s. planes.
Refinement. Refinement of *F*^2^ against ALL reflections. The weighted *R*-factor wR and goodness of fit *S* are based on *F*^2^, conventional *R*-factors *R* are based on *F*, with *F* set to zero for negative *F*^2^. The threshold expression of *F*^2^ > σ(*F*^2^) is used only for calculating *R*-factors(gt) etc. and is not relevant to the choice of reflections for refinement. *R*-factors based on *F*^2^ are statistically about twice as large as those based on *F*, and *R*- factors based on ALL data will be even larger.

### Fractional atomic coordinates and isotropic or equivalent isotropic displacement parameters (Å^2^)

**Table:**

	*x*	*y*	*z*	*U*_iso_*/*U*_eq_	Occ. (<1)
Co1	0.5000	0.5000	0.5000	0.03207 (11)	
O1	0.63648 (11)	0.55420 (6)	0.37193 (12)	0.0390 (3)	
O2	0.60938 (11)	0.55988 (6)	0.66433 (12)	0.0390 (3)	
N1	0.64813 (13)	0.63342 (7)	0.38754 (14)	0.0361 (3)	
N2	0.74874 (13)	0.55615 (7)	0.67045 (14)	0.0371 (3)	
N3	0.36144 (13)	0.59847 (8)	0.48907 (16)	0.0451 (4)	
N4	0.3061 (2)	0.85771 (11)	0.5359 (3)	0.0933 (7)	
N5	0.0012 (2)	0.71277 (15)	0.2587 (3)	0.1017 (8)	
C1	0.57248 (18)	0.68142 (10)	0.29627 (19)	0.0447 (4)	
H1	0.5131	0.6595	0.2229	0.054*	
C2	0.5829 (2)	0.76308 (11)	0.3113 (2)	0.0519 (5)	
H2	0.5295	0.7964	0.2487	0.062*	
C3	0.6710 (2)	0.79551 (10)	0.4175 (2)	0.0524 (5)	
H3	0.6780	0.8508	0.4283	0.063*	
C4	0.74983 (19)	0.74506 (10)	0.5088 (2)	0.0476 (4)	
H4	0.8123	0.7664	0.5802	0.057*	
C5	0.73654 (16)	0.66330 (9)	0.49468 (18)	0.0374 (4)	
C6	0.81807 (16)	0.60621 (9)	0.58911 (18)	0.0394 (4)	
C7	0.96264 (18)	0.60478 (12)	0.6027 (2)	0.0591 (5)	
H7	1.0118	0.6399	0.5493	0.071*	
C8	1.0347 (2)	0.55240 (13)	0.6937 (3)	0.0707 (6)	
H8	1.1323	0.5515	0.7019	0.085*	
C9	0.9616 (2)	0.50173 (12)	0.7720 (3)	0.0643 (6)	
H9	1.0092	0.4653	0.8333	0.077*	
C10	0.8172 (2)	0.50427 (9)	0.7606 (2)	0.0494 (5)	
H10	0.7673	0.4701	0.8153	0.059*	
C11	0.29971 (16)	0.65579 (10)	0.45990 (19)	0.0400 (4)	
C12	0.22334 (18)	0.72533 (10)	0.4249 (2)	0.0471 (4)	
C13	0.2679 (2)	0.79855 (12)	0.4860 (3)	0.0598 (5)	
C14	0.1018 (2)	0.71979 (11)	0.3308 (2)	0.0607 (5)	
O3	0.6888 (5)	0.5258 (4)	0.0838 (8)	0.0781 (13)	0.63
H3A	0.6797	0.4749	0.0780	0.117*	0.63
H3B	0.6715	0.5385	0.1685	0.117*	0.63
O3B	0.6196 (10)	0.5240 (9)	0.0694 (16)	0.108 (4)	0.37
H3C	0.6343	0.4776	0.0362	0.162*	0.37
H3D	0.6149	0.5187	0.1591	0.162*	0.37

### Atomic displacement parameters (Å^2^)

**Table:**

	*U*^11^	*U*^22^	*U*^33^	*U*^12^	*U*^13^	*U*^23^
Co1	0.03347 (17)	0.02267 (16)	0.03967 (19)	−0.00084 (11)	0.00116 (12)	−0.00023 (12)
O1	0.0486 (6)	0.0276 (5)	0.0414 (6)	−0.0048 (5)	0.0064 (5)	−0.0029 (5)
O2	0.0377 (6)	0.0376 (6)	0.0418 (6)	−0.0045 (5)	0.0037 (5)	−0.0035 (5)
N1	0.0404 (7)	0.0305 (7)	0.0378 (8)	−0.0036 (5)	0.0055 (6)	0.0028 (6)
N2	0.0399 (7)	0.0321 (7)	0.0380 (8)	−0.0036 (6)	−0.0035 (6)	−0.0019 (6)
N3	0.0413 (8)	0.0309 (7)	0.0620 (10)	0.0021 (6)	−0.0015 (7)	−0.0011 (7)
N4	0.1096 (17)	0.0452 (11)	0.1228 (19)	0.0050 (10)	−0.0019 (14)	−0.0175 (12)
N5	0.0858 (15)	0.0982 (16)	0.1116 (19)	0.0178 (12)	−0.0413 (15)	0.0107 (13)
C1	0.0485 (10)	0.0441 (10)	0.0410 (10)	−0.0025 (7)	0.0006 (8)	0.0083 (8)
C2	0.0592 (11)	0.0422 (10)	0.0548 (12)	0.0059 (8)	0.0086 (9)	0.0175 (9)
C3	0.0689 (12)	0.0290 (9)	0.0611 (13)	−0.0025 (8)	0.0147 (10)	0.0080 (8)
C4	0.0546 (11)	0.0361 (9)	0.0522 (11)	−0.0107 (8)	0.0053 (8)	−0.0011 (8)
C5	0.0375 (8)	0.0324 (8)	0.0421 (9)	−0.0057 (6)	0.0030 (7)	0.0019 (7)
C6	0.0389 (9)	0.0327 (8)	0.0457 (10)	−0.0061 (6)	−0.0015 (7)	0.0003 (7)
C7	0.0401 (10)	0.0592 (12)	0.0771 (15)	−0.0072 (9)	0.0000 (9)	0.0098 (11)
C8	0.0439 (11)	0.0753 (15)	0.0897 (17)	0.0017 (10)	−0.0113 (11)	0.0099 (13)
C9	0.0636 (14)	0.0550 (12)	0.0692 (15)	0.0087 (10)	−0.0214 (11)	0.0057 (10)
C10	0.0594 (11)	0.0387 (10)	0.0475 (11)	−0.0013 (8)	−0.0091 (9)	0.0075 (8)
C11	0.0370 (9)	0.0349 (9)	0.0481 (10)	−0.0006 (7)	0.0030 (7)	−0.0011 (7)
C12	0.0464 (10)	0.0353 (9)	0.0590 (12)	0.0099 (7)	0.0019 (9)	0.0029 (8)
C13	0.0659 (13)	0.0389 (11)	0.0748 (15)	0.0136 (9)	0.0073 (11)	−0.0003 (10)
C14	0.0626 (13)	0.0488 (11)	0.0688 (14)	0.0153 (9)	−0.0046 (11)	0.0099 (10)
O3	0.118 (4)	0.0587 (17)	0.058 (2)	0.012 (3)	0.015 (3)	−0.0030 (15)
O3B	0.164 (11)	0.086 (4)	0.075 (5)	0.034 (8)	0.018 (8)	−0.002 (4)

### Geometric parameters (Å, °)

**Table:**

Co1—O2	2.0503 (11)	C4—H4	0.9300
Co1—O2^i^	2.0503 (11)	C5—C6	1.478 (2)
Co1—O1	2.0691 (11)	C6—C7	1.378 (2)
Co1—O1^i^	2.0691 (11)	C7—C8	1.367 (3)
Co1—N3^i^	2.1093 (14)	C7—H7	0.9300
Co1—N3	2.1093 (14)	C8—C9	1.359 (3)
O1—N1	1.3346 (16)	C8—H8	0.9300
O2—N2	1.3318 (16)	C9—C10	1.378 (3)
N1—C1	1.340 (2)	C9—H9	0.9300
N1—C5	1.353 (2)	C10—H10	0.9300
N2—C10	1.342 (2)	C11—C12	1.396 (2)
N2—C6	1.350 (2)	C12—C14	1.400 (3)
N3—C11	1.145 (2)	C12—C13	1.401 (3)
N4—C13	1.140 (3)	O3—H3A	0.8567
N5—C14	1.134 (3)	O3—H3B	0.8584
C1—C2	1.374 (3)	O3—H3C	1.0384
C1—H1	0.9300	O3—H3D	1.0555
C2—C3	1.362 (3)	O3B—H3A	1.0017
C2—H2	0.9300	O3B—H3B	1.0464
C3—C4	1.379 (3)	O3B—H3C	0.8522
C3—H3	0.9300	O3B—H3D	0.8564
C4—C5	1.376 (2)		
			
O2—Co1—O2^i^	180.00 (5)	N1—C5—C4	118.94 (15)
O2—Co1—O1	85.58 (5)	N1—C5—C6	118.18 (13)
O2^i^—Co1—O1	94.42 (5)	C4—C5—C6	122.86 (15)
O2—Co1—O1^i^	94.42 (5)	N2—C6—C7	118.58 (16)
O2^i^—Co1—O1^i^	85.58 (5)	N2—C6—C5	118.84 (14)
O1—Co1—O1^i^	180.00 (5)	C7—C6—C5	122.49 (15)
O2—Co1—N3^i^	93.91 (5)	C8—C7—C6	120.92 (19)
O2^i^—Co1—N3^i^	86.09 (5)	C8—C7—H7	119.5
O1—Co1—N3^i^	86.63 (5)	C6—C7—H7	119.5
O1^i^—Co1—N3^i^	93.37 (5)	C9—C8—C7	118.97 (19)
O2—Co1—N3	86.09 (5)	C9—C8—H8	120.5
O2^i^—Co1—N3	93.91 (5)	C7—C8—H8	120.5
O1—Co1—N3	93.37 (5)	C8—C9—C10	120.15 (18)
O1^i^—Co1—N3	86.63 (5)	C8—C9—H9	119.9
N3^i^—Co1—N3	180.0	C10—C9—H9	119.9
N1—O1—Co1	114.83 (9)	N2—C10—C9	119.79 (17)
N2—O2—Co1	116.72 (9)	N2—C10—H10	120.1
O1—N1—C1	119.17 (13)	C9—C10—H10	120.1
O1—N1—C5	119.23 (12)	N3—C11—C12	179.43 (19)
C1—N1—C5	121.60 (14)	C11—C12—C14	118.80 (16)
O2—N2—C10	119.10 (14)	C11—C12—C13	119.73 (16)
O2—N2—C6	119.31 (12)	C14—C12—C13	121.46 (15)
C10—N2—C6	121.56 (15)	N4—C13—C12	179.0 (2)
C11—N3—Co1	166.34 (15)	N5—C14—C12	176.8 (3)
N1—C1—C2	119.85 (16)	H3A—O3—H3B	106.1
N1—C1—H1	120.1	H3A—O3—H3C	32.7
C2—C1—H1	120.1	H3B—O3—H3C	117.5
C3—C2—C1	120.34 (16)	H3A—O3—H3D	82.0
C3—C2—H2	119.8	H3B—O3—H3D	36.9
C1—C2—H2	119.8	H3C—O3—H3D	82.1
C2—C3—C4	118.89 (17)	H3A—O3B—H3B	84.0
C2—C3—H3	120.6	H3A—O3B—H3C	34.1
C4—C3—H3	120.6	H3B—O3B—H3C	117.3
C5—C4—C3	120.36 (17)	H3A—O3B—H3D	85.3
C5—C4—H4	119.8	H3B—O3B—H3D	37.3
C3—C4—H4	119.8	H3C—O3B—H3D	107.2

Symmetry codes: (i) −*x*+1, −*y*+1, −*z*+1.

### Hydrogen-bond geometry (Å, °)

**Table:**

*D*—H···*A*	*D*—H	H···*A*	*D*···*A*	*D*—H···*A*
O3—H3B···O1	0.86	2.00	2.851 (8)	173
O3B—H3D···O1	0.86	2.09	2.890 (15)	156
O3—H3A···N4^ii^	0.86	2.24	3.028 (8)	152
O3B—H3C···N4^ii^	0.85	2.21	3.056 (15)	174
C1—H1···N4^iii^	0.93	2.55	3.437 (3)	161
C4—H4···N5^iv^	0.93	2.38	3.287 (3)	165
C10—H10···N4^v^	0.93	2.48	3.390 (3)	164

Symmetry codes: (ii) −*x*+1, *y*−1/2, −*z*+1/2; (iii) *x*, −*y*+3/2, *z*−1/2; (iv) *x*+1, −*y*+3/2, *z*+1/2; (v) −*x*+1, *y*−1/2, −*z*+3/2.
